# Clinical and Molecular Spectrum of Glucose-6-Phosphate Isomerase Deficiency. Report of 12 New Cases

**DOI:** 10.3389/fphys.2019.00467

**Published:** 2019-05-07

**Authors:** Elisa Fermo, Cristina Vercellati, Anna Paola Marcello, Anna Zaninoni, Selin Aytac, Mualla Cetin, Ilaria Capolsini, Maddalena Casale, Sabrina Paci, Alberto Zanella, Wilma Barcellini, Paola Bianchi

**Affiliations:** ^1^UOC Ematologia, UOS Fisiopatologia delle Anemie, Fondazione IRCCS Ca’ Granda Ospedale Maggiore Policlinico di Milano, Milan, Italy; ^2^Department of Pediatric Hematology, Faculty of Medicine, Hacettepe University, Ankara, Turkey; ^3^Pediatric Oncohematology Section with BMT, Santa Maria della Misericordia Hospital, Perugia, Italy; ^4^Department of Woman, Child and General and Special Surgery, University of Campania “Luigi Vanvitelli”, Naples, Italy; ^5^Dipartmento di Pediatria, ASST Santi Paolo e Carlo, Presidio Ospedale San Paolo Universita’ di Milano, Milan, Italy

**Keywords:** red cell disorders, chronic hemolytic anemias, red cell metabolism, glucose-6-phosphate isomerase deficiency, glycolysis

## Abstract

Glucose-6-phosphate isomerase (GPI, EC 5.3.1.9) is a dimeric enzyme that catalyzes the reversible isomerization of glucose-6-phosphate to fructose-6-phosphate, the second reaction step of glycolysis. GPI deficiency, transmitted as an autosomal recessive trait, is considered the second most common erythro-enzymopathy of anaerobic glycolysis, after pyruvate kinase deficiency. Despite this, this defect may sometimes be misdiagnosed and only about 60 cases of GPI deficiency have been reported. GPI deficient patients are affected by chronic non-spherocytic hemolytic anemia of variable severity; in rare cases, intellectual disability or neuromuscular symptoms have also been reported. The gene locus encoding GPI is located on chromosome 19q13.1 and contains 18 exons. So far, about 40 causative mutations have been identified. We report the clinical, hematological and molecular characteristics of 12 GPI deficient cases (eight males, four females) from 11 families, with a median age at admission of 13 years (ranging from 1 to 51); eight of them were of Italian origin. Patients displayed moderate to severe anemia, that improves with aging. Splenectomy does not always result in the amelioration of anemia but may be considered in transfusion-dependent patients to reduce transfusion intervals. None of the patients described here displayed neurological impairment attributable to the enzyme defect. We identified 13 different mutations in the *GPI* gene, six of them have never been described before; the new mutations affect highly conserved residues and were not detected in 1000 Genomes and HGMD databases and were considered pathogenic by several mutation algorithms. This is the largest series of GPI deficient patients so far reported in a single study. The study confirms the great heterogeneity of the molecular defect and provides new insights on clinical and molecular aspects of this disease.

## Introduction

Glucose-6-phosphate isomerase (GPI, EC 5.3.1.9) is a dimeric enzyme that catalyzes the reversible isomerization of glucose-6-phosphate (G6P) to fructose-6-phosphate (F6P), the second reaction step of glycolysis (Kugler and Lakomek, 2000). In addition to the catalytic function of the dimeric enzyme, the monomeric form of GPI has been shown to act as a cytokine, its activities including neuroleukin (Gurney et al., 1986), myofibril-bound serine-protease inhibitor (Cao et al., 2000), autocrine motility factor (Watanabe et al., 1996), and the maturation and differentiation factor (Xu et al., 1996). More recently an unexpected relationship between GPI and phosphatidate phosphatase 1 (PAP1) activity involved in glycerolipid biosynthesis has also been reported (Haller et al., 2010).

Glucose-6-phosphate isomerase deficiency (OMIM 172400), transmitted as an autosomal recessive trait, is considered the second most common erythro-enzymopathy of anaerobic glycolysis, after pyruvate kinase deficiency. GPI deficient patients are affected by mild to severe chronic non-spherocytic hemolytic anemia (CNSHA); in rare cases intellectual disabilty or neuromuscular symptoms have also been reported (Van Biervliet et al., 1975; Kahn et al., 1978; Zanella et al., 1980; Schröter et al., 1985; Shalev et al., 1993; Jamwal et al., 2017).

The gene locus encoding GPI is located on chromosome 19q13.1 and contains 18 exons (Walker et al., 1995). So far, about 60 cases of GPI deficiency have been described, and more than 40 mutations have been reported at the nucleotide level (Kugler and Lakomek, 2000; Clarke et al., 2003; Repiso et al., 2006; Zhu et al., 2015; Manco et al., 2016; Jamwal et al., 2017; Zaidi et al., 2017; Kedar et al., 2018; Mojzikova et al., 2018). Missense mutations are the most common, but non-sense and splicing mutations have also been observed.

In this paper we report the clinical and molecular characterization of 12 patients affected by GPI deficiency: six new mutations of the *GPI* gene have been found and related to the clinical pattern. Long term follow-up allowed us to describe the clinical spectrum of the GPI deficiency from infancy to adulthood.

## Patients and Methods

### Patients

Twelve patients (eight males and four females) from 11 families, with a median age at admission of 13 years (ranging from 1 to 51) were studied; eight were of Italian origin, two were Turkish, one from Pakistan and one from Romania.

### Hematological and Enzyme Assays

Blood samples were collected after obtaining written informed consent from the patients and approval from the Institutional Ethical Committee. For patients under the age of 18, written informed consent was obtained from the parents. All the diagnostic procedures and investigations were performed in accordance with the Helsinki Declaration of 1975. Routine hematological investigations were carried out according to Dacie and Lewis (2001): complete blood count, reticulocyte count, bilirubin, serum ferritin levels, screening for abnormal/unstable hemoglobins, direct antiglobulin test. To exclude red cell membrane disorders, RBC morphology and red cell osmotic fragility tests were evaluated in all cases. When possible EMA binding tests (Bianchi et al., 2012), red cell protein content by SDS–PAGE analyses (Mariani et al., 2008), and RBC deformability analyses by LoRRca MaxSis (Laser-Assisted Optical Rotational Cell Analyzer, Mechatronics, NL) (Zaninoni et al., 2018) were performed. RBC enzymes activities were determined according to Beutler et al. (1977). The diagnosis of GPI deficiency was made through the exclusion of the most common causes of hemolytic anemia, by the demonstration of a reduced GPI activity in the probands or in the parents, and by the identification of homozygous or compound heterozygous mutations in the *GPI* gene.

### Molecular Analysis

Genomic DNA was extracted from leukocytes collected from peripheral blood, using standard manual methods (Sambrook et al., 1989). The entire codifying region and intronic flanking regions of the *GPI* gene were analyzed by direct sequencing (ABI PRISM 310 Genetic Analyzer, Applied Biosystems, Warrington, United Kingdom) using the Big Dye Terminator Cycle Sequencing Kit (Applied Biosystems, Warrington, United Kingdom).

When available, total RNA was isolated from leucocytes using TRIzol (Life Technologies, Paisley, United Kingdom) and reverse transcribed to cDNA using random hexamer primers and AMV reverse transcriptase. The entire GPI cDNA was amplified by PCR and automatically sequenced. (RefSeq: ENST00000356487, UniProt P06744). **Table 1** reports the primers used for molecular analysis.

**Table 1:** Primers used for DNA analysis of GPI gene.

1F	CGCCCACGCGCCTCGCT	1R	GCCCCCGCCTCCAGACC
2F	TCTTCTGGGAACAGCTCCTG	2R	GAGGAGGTGACTGAGGTCTA
3F	CGTCTGTCTGTCTCATTGGG	3R	GGTGAAGACACAGGGTGATG
4F	TGTCTAGTGGATAGAGGGCC	4R	CCCCTCCCTTAAGCTGCA
5F	CCAGGACACGGCAGTAATGA	5R	ACAGCCAGGTCCCATCCCTG
6F	GTCTGGGCACTGTTGGTCC	6R	CCAAAAGGGACCAATGGCCA
7F	GTCACTGTCACTGACCTGCA	7R	CCGCCTTCACTTCCAACTTC
8F	CTCAGAACCAAGGACTGGGA	8R	ATCCACCAGACCTACGAACC
9F	TCACGGAGCACAGCTCCCT	9R	GCTAGGTATGCAGCAGGTAC
10F	GTGCAAGACCAGGGACAGG	10R	GCATGATGTTCAGGGACACAA
11F	GCCTTCCTTCGTTGCAGAAG	11R	GCAGGATGAGTGGGAGCTG
12F	CTCTGCCAAGTGCTGGCCA	12R	AATGGGGCAAAGAGCTCCTG
13F	TTACAGGCTTGAGCCACTGC	13R	ACTGTGGTCACCCACATGAC
14F	GGAGGGAAAGGATCTTCCAG	14R	GCCAACCAATGCACCAGGTT
15F	GAAGTACCAGGCGGTCTTGT	15R	CCCATTCTGTAGGACAAGCC
16F	ACCTGCACGTCTCAGCCTC	17R	GTGGTATGAGGAAGGCTCTAA
18F	TAGGGGAGGGCCGGGAATA	18R	CCACAACCAGAGGGTGCTC


To clarify the pathogenetic effect of the genotype identified in patient seven and to exclude other concomitant causes of hemolysis, the DNA sample of the patient was further analyzed on an NGS-targeted panel designed by SureDesign software (Agilent Technologies, Santa Clara, CA, United States), containing 40 genes associated with congenital hemolytic anemias. Libraries were obtained by the HaloPlexHS Target Enrichment System Kit and sequenced on a MiSeq platform (Illumina, San Diego, CA, United States).

## Results

**Table 2** reports the main clinical and hematological data in the 12 GPI deficient patients at the time of diagnosis. In 10/12 patients, extensive clinical data, family history, and laboratory data were available, with a median follow-up of 18 years (ranging from 2 to 40 years).

**Table 2:** Clinical and hematological data in the 12 GPI deficient patients at the time of the diagnosis.

Pt	Age	Sex	Neon. Jaun.	Extx	Tx	Tot. n.	Splenect. (age)	Colecyst. (age)	Hb g/dL	Retics 10^9^/L	Hb g/dL	Retics 10^9^/L	MCV (fL)	Unc. Bil (mg/dL)	SF (ng/mL)
									**Pre-splenectomy**		**Post-splenectomy**				
1	2	M	Yes	No	Occasional	4	No	No	6.1ˆ-10.2	231	–	–	103	1.1	n.a.
2	6	F	No	No	Occasional	2	No	No	6.2ˆ-11.6	166	–	–	94.9	n.a.	n.a.
3	40	M	Yes	Yes	Occasional	n.a.	Yes (9)	No	n.a.	n.a.	11.5	445	126.5	3.56	2356
4	8	F	No	No	Occasional	n.a.	Yes (7)	Yes (7)	9.4	113	10	364	105.1	3–11.9	488
5	1	M	Yes	Yes	Occasional	10	No	No	10	n.a.	–	–	102	5	202
6	51	F	No	no	Occasional	9	Yes (17)	Yes (18)	n.a.	n.a.	10.5	170	119	3,18	353
7	3	M	No	No	Occasional	n.a.	No	No	11.7	347	–	–	127.8	0.93	210
8	1	M	Yes	Yes (2)	Regular (4w)	n.a.	No	No	8.5	410	–	–	84.8	2	n.a.
9	18	F	No	No	Regular^∗^ (4w)	>50	Yes (6)	Yes	5.4ˆ-8.9	210	9.4	1420	127	13.4 (post)	1123
10	23	M	No	No	Regular^∗^ (4w)	>30	Yes (3)	Yes	2.7ˆ-8.4	660	9.2	1740	123	8.2 (post)	185
11	18	M	No	No	No	0	No	No	10.8	200	–	–	103.3	4.3	n.a.
12	46	M	Yes	No	No	0	Yes (45)	No	8.0	126	13.9	342	101.6	2.7	n.a.
Ref. values									12–16	16–84	12–16	16–84	78–99	<0.75	30–400


*Tx, transfusions; SF, serum ferritin; n.a., not available; ^∗^, case 9: every 4 weeks until splenectomy then occasional; case 10 every 4 week until splenectomy; ˆ= during hemolytic crises.*

**FIGURE 1:**
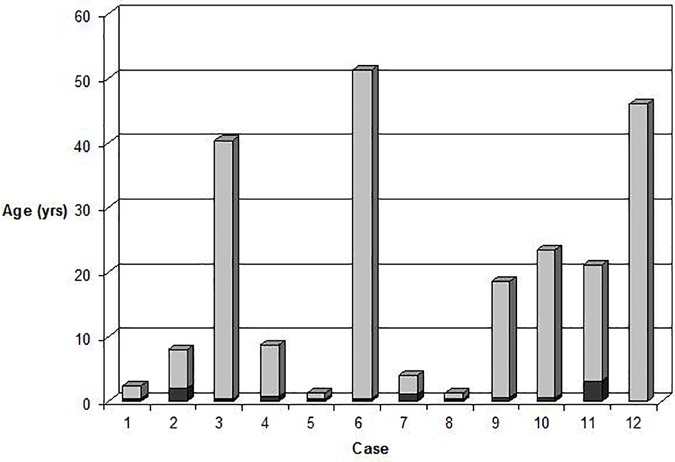
Age at onset of anemia (dark gray) compared with age at diagnosis (light gray) in the 12 GPI deficient patients.

Consanguinity was confirmed in one Turkish patient and suspected in another two families originating from small Italian villages. Despite the onset of anemia reported at birth or early infancy, the median age of diagnosis varied greatly, half of the cases in fact were diagnosed in adulthood (18 to 51 years) (**Figure 1**). Six patients were misdiagnosed before receiving the correct diagnosis of GPI in this study: the most common diagnostic errors were hereditary spherocytosis (four cases), thalassemia (one case), or G6PD deficiency (one case). None of the patients showed neurological symptoms attributable to GPI deficiency. Growth and intellectual disability were reported only in case 5, affected by phenylketonuria’ (PKU), untreated during infancy. Case 12 had a concomitant G6PD deficiency (0.6 IU/gHb; ref. ranges: 7.2–9.6).

All the patients displayed chronic macrocytic anemia before splenectomy, with median Hb levels during follow-up of 9.4 g/dL (range 8–11.3); median VGM 119 fL (84.8–127.8), MCHC 32.1 g/dL (28.6–33), increased absolute reticulocyte number (210 × 10^9^/L, range 113–660) and increased unconjugated bilirubin. Recurrent drastic drops down of Hb levels (median 5.4 g/dL, 2.7–6.2) were reported during infection/aplastic crisis in five patients.

In 8/12 cases, information on iron status was available, serum ferritin levels were increased in most patients (median 353 ng/mL, 90–2356), two of them requiring chelation therapy due to iron overload.

**FIGURE 2:**
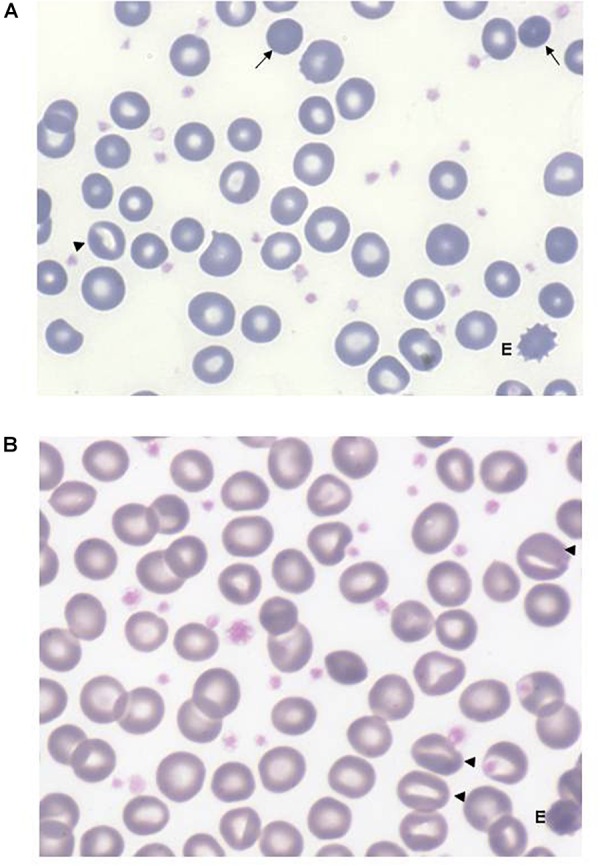
Peripheral red cell morphology from a non-splenectomized **(A)** and from a splenectomized **(B)** GPI patient (May-Grünwald’s Giemsa staining). Anisopoikilocytosis with presence of rare spherocytes (arrows), stomatocytes (triangles), more evident after splenectomy, rare echinocytes (E). The increased platelet number in panel **(B)** is due to splenectomy, some large and vacuolated platelets are likely due to EDTA anticoagulant.

**FIGURE 3:**
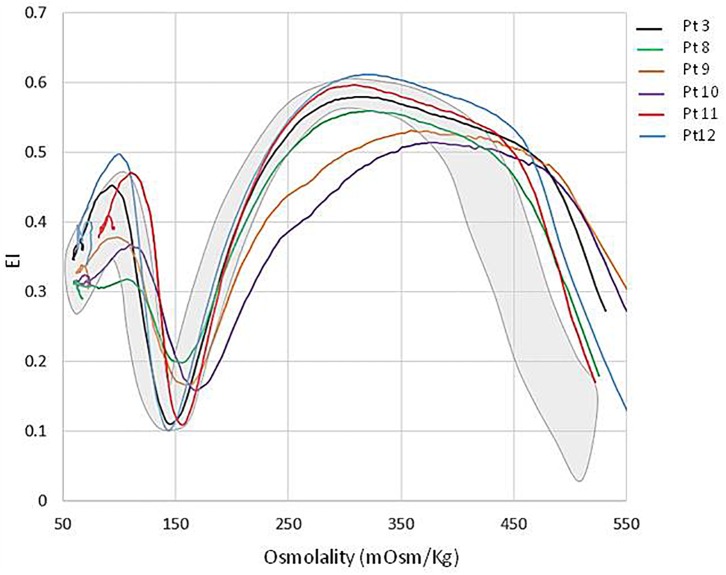
LoRRca Osmoscan curve of 6 GPI deficient patients compared with normal controls (gray area).

All the patients displayed a normal osmotic fragility and EMA-binding test. RBC morphology, available in 9/12 patients, was unremarkable although not comparable to normal subjects; a few spherocytes, stomatocytes (ranging from 3 to 10%), echinocytes (3 to 4%), rare ovalocytes or target cells were reported. A more compromised RBC morphology was observed in the splenectomized patients (**Figure 2**). In six patients, RBC deformability was investigated by LoRRca Osmoscan analysis. Interestingly, all the them showed an altered enlarged Osmoscan curve associated with significantly increased Omin (median 156, range 126–176, *p* < 0.001) and, even more, Ohyper (median 527, range 439–579, *p* < 0.001). EImax and AUC values were decreased compared to the controls (**Figure 3**).

All the patients but one displayed a reduced GPI activity (from 10 to 40% of low normal reference range). In case 8, who showed normal GPI activity, the diagnosis was reached by studying the GPI activity in the parents.

### Glucose-6-Phosphate Isomerase Deficiency in Infancy

No intrauterine growth retardation and/or fetal distress were observed in this series of patients prior to birth. Information on the neonatal period was available in 11 patients. Five of them displayed anemia at birth, the remaining six in early infancy (all before 3 years of age). Neonatal jaundice was present in five patients, three of whom required exchange transfusion. During childhood all patients but two needed blood transfusions: three of them regularly with a transfusion interval of 4 to 8 weeks. The other patients were occasionally transfused in concomitance of hemolytic crises due to infections.

### Glucose-6-Phosphate Isomerase Deficiency and Splenectomy

Six patients were splenectomized, all of them before the diagnosis of GPI deficiency. Only one patient recovered from anemia; in the remaining cases, although resulting only in a slight increase of Hb levels (0.5–1 g/dL), splenectomy greatly reduced or even eliminated transfusion requirement.

Interestingly, in some patients a considerable increase of the reticulocyte counts and unconjugated bilirubin was observed after splenectomy. No thrombotic events have been reported in the six splenectomized patients, since their surgeries.

### Molecular Heterogeneity of Glucose-6-Phosphate Isomerase Deficiency

**Table 3** reports the biochemical and molecular data of the GPI deficient patients.

**Table 3:** Biochemical and molecular data of the GPI deficient patients.

Pt	GPI activity (IU/gHb)	Residual activity %	Mutation	Effect
1	6	10%	**c.145G>C/c.921C>A**	**p.Gly49Arg/p.Phe307Leu**
2	10.5	19%	**c.311 G>A/**c.584C>T	**p.Arg104Gln**/p.Thr195Ile
3	18	32%	**c.307C>G/c.307C>G**	**p.Leu103Val/p.Leu103Val**
4	15.7	28%	c.301G>A/c.1009G>A	p.Val101Met/p.Ala337Thr
5	13.3	24%	c.1009G>A/c.1009G>A	p.Ala337Thr/p.Ala337Thr
6	14.3	26%	c.584C>T/c.584C>T	p.Thr195Ile/p.Thr195Ile
7	16	29%	c.489A>G (rs1801015)/c.1415G>A	LOH/p.Arg472His
8	54.6	98%	**c.269T>C/**c.1066G>A	**p.Ile90Thr/**p.Asp356Asn
9	22	40%	c.1040G>A/c.1040G>A	p.Arg347His/p.Arg347His
10	17	30%	c.1040G>A/c.1040G>A	p.Arg347His/p.Arg347His
11	20.2	36.5%	**c.839T>G/c.839T>G**	**p.Ile280Ser/p.Ile280Ser**
12	4.8	9%	c.1574 T>C/c.1574 T>C	p. Ile525Thr/p.Ile525Thr
Ref. range	55,3–72,3			


*Percentage of residual GPI activity was calculated on the lower reference value. New mutations are reported in bold.*

**FIGURE 4:**
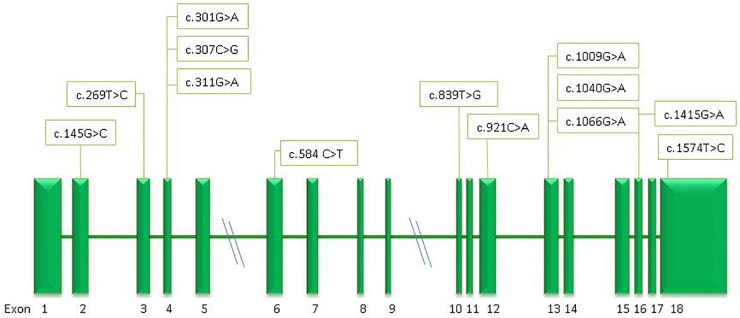
Schematic representation of the *GPI* gene and position of the mutations identified in this study.

Thirteen different missense mutations were found in the GPI gene, six of them never described before (c.145G>C, p.Gly49Arg; c.269T>C, p.Ile90Thr; c.307C>G, p.Leu103Val; c.311 G>A, p.Arg104Gln; c.839T>G, p.Ile280Ser; c.921C>A, p.Phe307Leu) (**Figure 4**).

All the new mutations affect highly conserved residues, and were predicted to have pathogenic effects by Polyphen-2, Mutation Taster, and M-CAP (**Table 4**).

**Table 4:** List of new variants identified.

HGVS coding	HGVS protein	GPI structure	Exon	Status	Polyphen-2	M-CAP	MAF1000G	MAF ExAC	RefSeqID
c.145G>C	Gly49Arg	Lβ1-β2	2	Het	1.000 (0.00; 1.00) D	0.358 P	–	–	–
c.269T>C	p.Ile90Thr	Lα6-Turn	3	Het	1.000 ( 0.00; 1.00) D	0.343 P	–	–	–
c.307C>G	p.Leu103Val	α8	4	Hom	0.649 (0.87; 0.91) PD	0.479 P	–	–	–
c.311 G>A	p.Arg104Gln	Lα8-b4	4	Het	0.544 (0.88; 0.91) PD	0.577 P	–	–	–
c.839T>G	p.Ile280Ser	α21	10	Hom	1.000 (0.00; 1.00) D	0.611 P	–	–	–
c.921C>A	p.Phe307Leu	α23	12	Het	1.000 (0.00; 1.00) D	0.383 P	–	*A* = 0.000008	rs754782152


*Polyphen-2 analysis (HumDiv) PD, possibly damaging; D, probably damaging; in bracket sensitivity, specificity; M-CAP P, possibly pathogenetic (ref. Jagadeesh et al., 2016). Recommended pathogenicity threshold: Polyphen-2 > 0.8 (with misclassified pathogenetic variants 31%); M-CAP > 0.025 (with misclassified pathogenetic variants 5%).*

Seven patients were homozygote and four compounds heterozygotes for two different mutations. In patient 7, despite the sequencing of the entire *GPI* codifying region, intronic flanking regions and promoter, we were able to find only one mutation at the heterozygous level (p.Arg472His), transmitted by the mother. In addition, we detected the polymorphism c.489A>G [synonymous variant p.Gly163=, rs1801015, GMAF 0.20070 (G), ExAC 0.11116], transmitted by the father. No other pathogenetic mutations were detected by the NGS targeted sequencing of 40 genes associated with congenital hemolytic anemias, confirming that *GPI* deficiency was the only cause of anemia in this patient. cDNA analysis in the proband and his parents revealed a loss of heterozygosity, with only the maternal allele present at the cDNA level, suggesting that the paternal allele was not expressed or rapidly degraded. Despite this, we did not find a difference in clinical severity in these patients with respect to the other GPI patients carrying two missense mutations.

## Discussion

The present cohort of GPI deficient patients represents the largest series so far described in a single study, collecting retrospective information and follow-up data over a median period of 18 years. All the cases were never reported before, consistently increasing the number of GPI patients reported in literature (Kugler and Lakomek, 2000; Clarke et al., 2003; Repiso et al., 2006; Warang et al., 2012; Adama van Scheltema et al., 2015; Zhu et al., 2015; Manco et al., 2016; Jamwal et al., 2017; Zaidi et al., 2017; Burger et al., 2018; Kedar et al., 2018; Mojzikova et al., 2018).

Despite the fact that GPI deficiency is considered the second most frequent RBC enzymopathy of anaerobic glycolysis after pyruvate kinase, the exact frequency of this disorder is not known and a diagnosis is often difficult to reach; this may be due to the lack of availability of the enzymatic assay, performed only in a few specialized centers, or because of the lack of knowledge about some rare disorders for which specific tests are not considered during laboratory investigations (Bianchi et al., 2018; Kedar et al., 2018; Sonaye et al., 2018). Moreover, due to the similarity in clinical presentation with other congenital hemolytic anemias, an exact diagnosis is often delayed.

An increasing number of new diagnoses might be expected in the coming years due to the advent of new NGS technologies that allow the simultaneous analysis of multiple genes associated to rare/very rare hemolytic anemias. At least three additional GPI-deficient patients have been reported in the literature in the last 2 years using these technologies (Jamwal et al., 2017; Kedar et al., 2018; Russo et al., 2018).

The possibility to evaluate a consistent group of patients from infancy to adulthood allowed us to describe the clinical picture of GPI deficiency, which is characterized by the onset of chronic macrocytic anemia at birth or early infancy, reticulocytosis, jaundice and splemomegaly associated with mild hepatomegaly; in all the patients in which the information was available, pregnancy was uneventful with normal growth development.

This clinical pattern is in line with cases previously described by our group (Baronciani et al., 1996); however, in some patients a more severe clinical presentation, i.e., hydrops fetalis, has been reported (Ravindranath et al., 1987; Adama van Scheltema et al., 2015).

Neuromuscular impairment or mental retardation are rare complication sometimes reported in GPI deficiency (Van Biervliet et al., 1975; Kahn et al., 1978; Zanella et al., 1980; Shalev et al., 1993; Kugler et al., 1998; Jamwal et al., 2017), as well as in other glycolytic enzyme defects caused by ubiquitously expressed genes (i.e., phosphoglycerate kinase deficiency, phosphofructokinase deficiency or triosephosphate isomerase deficiency). The link between GPI deficiency and neuromuscular dysfunction has not been fully established, and has been attributed to the fact that the monomeric form of GPI is identical to neuroleukin (NLK), a neurotrophic factor that supports the survival of embryonic spinal neurons, skeletal neurons and sensory neurons; however, the proposed hypothesis on the molecular mechanism leading to a neuromuscular dysfunction are in some cases contradictory (Kugler et al., 1998; Repiso et al., 2005). Actually, only three GPI deficient cases with neurological impairment were characterized at molecular level: two of them were homozygous for mutations p.Arg347Cys and p.Arg347His, respectively, and one was compound heterozygous for mutations p.His20Pro and p.Leu339Pro (Beutler et al., 1997; Kugler et al., 1998; Jamwal et al., 2017). A large number of cases with mutations affecting amino acid Arg347 (including two in our series) have been reported with only hematological involvement, suggesting that other possible confounding factors, independent from enzyme deficiency itself, such as kernicterus (Jamwal et al., 2017) or other genetic defects in consanguineous families, may contribute to the clinical phenotype.

A long follow-up time allowed us to shine light on other possible features of GPI deficiency not yet clearly reported in literature: (a) increased sensitivity to infections that result in a dramatic drop-down of hemoglobin levels persisting also in adults, (b) a low response to splenectomy resulting only in a slight increase of Hb levels, however eliminating or reducing the transfusion requirement in all patients, (c) a tendency to increase the reticulocyte number after splenectomy, probably due to selective sequestration of younger GPI defective erythrocytes by the spleen as previously hypothesized in PK deficiency (Mentzer et al., 1971; Matsumoto et al., 1972).

Increased sensitivity to infections was reported in other GPI cases (Helleman and Van Biervliet, 1976; Repiso et al., 2006; Manco et al., 2016; Kedar et al., 2018), making this aspect relevant in the follow-up, suggesting that adequate vaccination coverage should be considered.

As previously reported, GPI-deficient red cells produce an altered Osmoscan profile (LoRRca analyzer), characterized by a right enlarged opened curve (Zaninoni et al., 2018). These findings, which result in a statistically significative increase of Ohyper values, offer an initial laboratory screen for patients with this rare enzyme defect. A possible explanation may reside in an increased red cell volume, or a cellular overhydrated state resulting in cell swelling of an origin not yet investigated.

Increased thrombotic risk after splenectomy, clearly demonstrated in hereditary xerocytosis, and in overhydrated stomatocytosis (Fermo et al., 2017; Iolascon et al., 2017) has recently been reported in some enzyme defects i.e., pyruvate kinase deficiency) (Grace et al., 2018). No thrombotic events have been reported in the analyzed series, or in the GPI deficient cases reported in literature; however, we cannot exclude that this information might be lost at follow up.

Information on iron status and erythropoietic activity in GPI deficiency is scant, although it is known that iron overload may frequently occur in other more common glycolytic enzymopathies as a consequence of various factors, including hyperhaemolysis or ineffective erythropoiesis. Only iron stores (ferritin levels) were available in the present series and found to be elevated in four of seven patients, underlying the need of monitoring iron status in this disease.

Glucose-6-phosphate isomerase deficiency shows a wide molecular heterogeneity with more than 40 mutations in the GPI gene currently listed in the Human Gene Mutation Database^1^. Most of them are missense, covering about 93% of the total mutations identified, with only a few splicing, nonsense or frameshift mutations (Kugler and Lakomek, 2000; Manco et al., 2016). This is in line with the findings in our series, where all the different mutations identified were missense. Despite this, loss of heterozygosity at the cDNA level in patient 7, who did not show the second causative mutation, neither by Sanger sequencing nor by NGS targeted sequencing, may suggest that in GPI deficiency some drastic molecular abnormalities escape the conventional screening techniques. Interestingly, patient 7 carried the paternal allele on the silent polymorphic variant c.489A>G (p.Gly163=), that is located in the third nucleotide of exon 6. Although we did not perform functional *in vitro* analysis of this silent mutation, we cannot exclude that the variant, although polymorphic, may interfere with the normal splicing, resulting in an unstable mRNA, rapidly degraded.

Despite the molecular heterogeneity, some recurrent mutations have been identified in GPI deficiency. This is the case of missense mutations affecting the amino acid Arg347, here detected in two brothers of Turkish origin (c.1040G>A, p.Arg347His) and already reported in literature in other unrelated patients of different ethnical origins (Walker et al., 1993; Repiso et al., 2006; Lin et al., 2009); another mutation at the same codon (c.1039 C>T, p. Arg347Cys) has also been described (Xu and Beutler, 1994; Lin et al., 2009), suggesting the presence of a mutational hotspot (Repiso et al., 2005). Arg347 is a highly conserved residue (GERP 5.65), falling in the region responsible for GPI dimerization. It has been hypothesized that a mutation in these residues causes a loss of GPI capability to dimerize, making the enzyme more susceptible to thermolability; actually, kinetic studies performed in a mutant enzyme from an homozygous p.Arg347His patient, showed that the Km for G6P and for F6P were not altered, but the thermostability was drastically reduced (Repiso et al., 2005).

Different than expected and previously reported in literature (Walker et al., 1993; Repiso et al., 2005) the cases in this series carrying mutation p.Arg347His did not show a drastic reduction of GPI activity (30–40% of residual activity vs. 18% reported by others); this could be explained by technical variability in the enzymatic assay, or by the very high number of reticulocytes found in our patients at the time of the assay, which may display an higher enzyme activity than mature red cells (Beutler et al., 1977). Other recurrent mutations found in this series were p.Thr195Ile and p.Val101Met, already reported in Italian patients by Baronciani et al. (1996).

## Conclusion

In conclusion, the study confirms the great heterogeneity of the molecular defect and provides new insights on clinical and molecular aspects of this disease.

## Author Contributions

EF and PB performed the molecular analysis, analyzed the results, prepared and revised the manuscript. CV, AM, and AnZ performed the hematologic and biochemical investigations. SA, MuC, IC, MaC, SP, AlZ, and WB performed the patient follow-up and revision of the manuscript.

## Conflict of Interest Statement

The authors declare that the research was conducted in the absence of any commercial or financial relationships that could be construed as a potential conflict of interest.

## Footnotes

1 www.hgmd.cf.ac.uk
